# Utilization of non-invasive ventilation before prehospital emergency anesthesia in trauma – a cohort analysis with machine learning

**DOI:** 10.1186/s13049-025-01350-1

**Published:** 2025-03-03

**Authors:** André Luckscheiter, Manfred Thiel, Wolfgang Zink, Johanna Eisenberger, Tim Viergutz, Verena Schneider-Lindner

**Affiliations:** 1 Medical Faculty Mannheim, Heidelberg University, Mannheim, Germany; 2 Department of Anesthesiology, Operative Intensive Care Medicine and Emergency Medicine, Ludwigshafen City Hospital, Bremserstrasse 79, 67063 Ludwigshafen, Germany; 3 Department of Anesthesiology and Surgical Intensive Care Medicine, University Medical Centre Mannheim, Mannheim, Germany; 4 Centre for Quality Management in Emergency Medical Service Baden-Wuerttemberg (SQR-BW), Stuttgart, Germany; 5 Department of Anesthesiology, Intensive Care and Pain Therapy, BG Trauma Centre Tuebingen, Tuebingen, Germany

**Keywords:** Intubation, intratracheal*, Machine learning*, Trauma, Decision trees, Bayes theorem, Non-invasive ventilation

## Abstract

**Background:**

For preoxygenation, German guidelines consider non-invasive ventilation (NIV) as a possible method in prehospital trauma care in the absence of aspiration, severe head or face injuries, unconsciousness, or patient non-compliance. As data on the utilization and characteristics of patients receiving NIV are lacking, this study aims to identify predictors of NIV usage in trauma patients using machine learning and compare these findings with the current national guideline.

**Methods:**

A cross-regional registry of prehospital emergency services in southwestern Germany was searched for cases of emergency anesthesia in multiply injured patients in the period from 2018 to 2020. Initial vital signs, oxygen saturation, respiratory rate, heart rate, systolic blood pressure, Glasgow Coma Scale (GCS), injury pattern, shock index and age were examined using logistic regression. A decision tree algorithm was then applied in parallel to reduce the number of attributes, which were subsequently tested in several machine learning algorithms to predict the usage of NIV before the induction of anesthesia.

**Results:**

Of 992 patients with emergency anesthesia, 333 received NIV (34%). Attributes with a statistically significant influence (*p* < 0.05) in favour of NIV were bronchial spasm (odds ratio (OR) 119.75), dyspnea/cyanosis (OR 2.28), moderate and severe head injury (both OR 3.37) and the respiratory rate (OR 1.07). Main splitting points in the initial decision tree included auscultation (rhonchus and bronchial spasm), respiratory rate, heart rate, age, oxygen saturation and head injury with moderate head injury being more frequent in the NIV group (23% vs. 12%, *p* < 0.01). The rates of aspiration and the level of consciousness were equal in both groups (0.01% and median GCS 15, both *p* > 0.05). The prediction accuracy for NIV usage was high for all algorithms, except for multilayer perceptron and logistic regression. For instance, a Bayes Network yielded an AUC-ROC of 0.96 (95% CI, 0.95–0.96) and PRC-areas of 0.96 [0.96–0.96] for predicting and 0.95 [0.95–0.96] for excluding NIV usage.

**Conclusions:**

Machine learning demonstrated an excellent categorizability of the cohort using only a few selected attributes. Injured patients without severe head injury who presented with dyspnea, cyanosis, or bronchial spasm were regularly preoxygenated with NIV, indicating a common prehospital practice. This usage appears to be in accordance with current German clinical guidelines. Further research should focus on other aspects of the decision making like airway anatomy and investigate the impact of preoxygenation with NIV in prehospital trauma care on relevant outcome parameters, as the current evidence level is limited.

**Supplementary Information:**

The online version contains supplementary material available at 10.1186/s13049-025-01350-1.

## Background

Before and during invasive airway management, trauma patients are at high risk of hypoxia due to primary lung injury, hypovolemia, insufficient respiratory drive, lack of airway protection, or airway injury [1]. Therefore, counter-strategies are an important part of emergency airway management [2]. Basically, several methods are available: oxygen mask, bag-valve mask, high flow oxygen therapy or non-invasive ventilation (NIV) with positive end expiratory pressure. Whereas oxygen and bag-valve masks are inexpensive and easy to use, the amount of deliverable oxygen is limited and assisted respiratory support can be technically challenging [3, 4]. In contrast, NIV, especially in a pressure support mode, not only improves alveolar recruitment and therefore oxygenation and denitrogenation, but can also increase minute ventilation. This makes NIV a favored method of choice in hypercapnic respiratory failure [5, 6]. Baillard et al. demonstrated that, for the intubation of hypoxemic patients, preoxygenation with NIV is more effective at avoiding arterial oxyhemoglobin desaturation than a non-rebreather bag-valve mask [7]. In the emergency department and in the intensive care unit, Gibbs et al. recently reported not only a lower rate of cardiac arrest but also a halving of desaturation with NIV for preoxygenation compared to a simple oxygen mask. Importantly, the incidence of aspiration was not increased [8]. However, in prehospital emergency medicine, on-site invasive medical treatment might be challenging and available resources at the scene are limited. Furthermore, patients with medical emergencies often exhibit an altered state of consciousness or are at risk of aspiration, both of which are contraindications for NIV. Therefore, the current German guideline on prehospital airway management considers NIV only potentially superior in preoxygenation [2]. However, current data on preoxygenation methods for prehospital invasive airway management in Germany are lacking.

In recent years, studies using machine learning have given new insights in prehospital emergency care. Basically, machine learning can be applied to four different problems: earlier disease identification (for example forecasting resuscitation during transport), disease evolution prediction (e.g. success of resuscitation), disease phenotyping (like sepsis patterns) and guiding clinical decisions (e.g. airway management in trauma patients) [9–13]. In addition to a classical direct statistical attribute comparison, the algorithms of machine learning can gain deeper insights by addressing complex attribute dependencies [14].

The aim of the study is to use machine learning to identify and describe a cluster of severely injured patients that are treated with non-invasive ventilation before prehospital emergency anesthesia, based on a large subset of a nationwide emergency dataset. From this cluster, characteristics of patients receiving non-invasive ventilation and a description of the current utilization of NIV as a preoxygenation technique in prehospital trauma care shall be derived.

## Methods

**Fig. 1:**
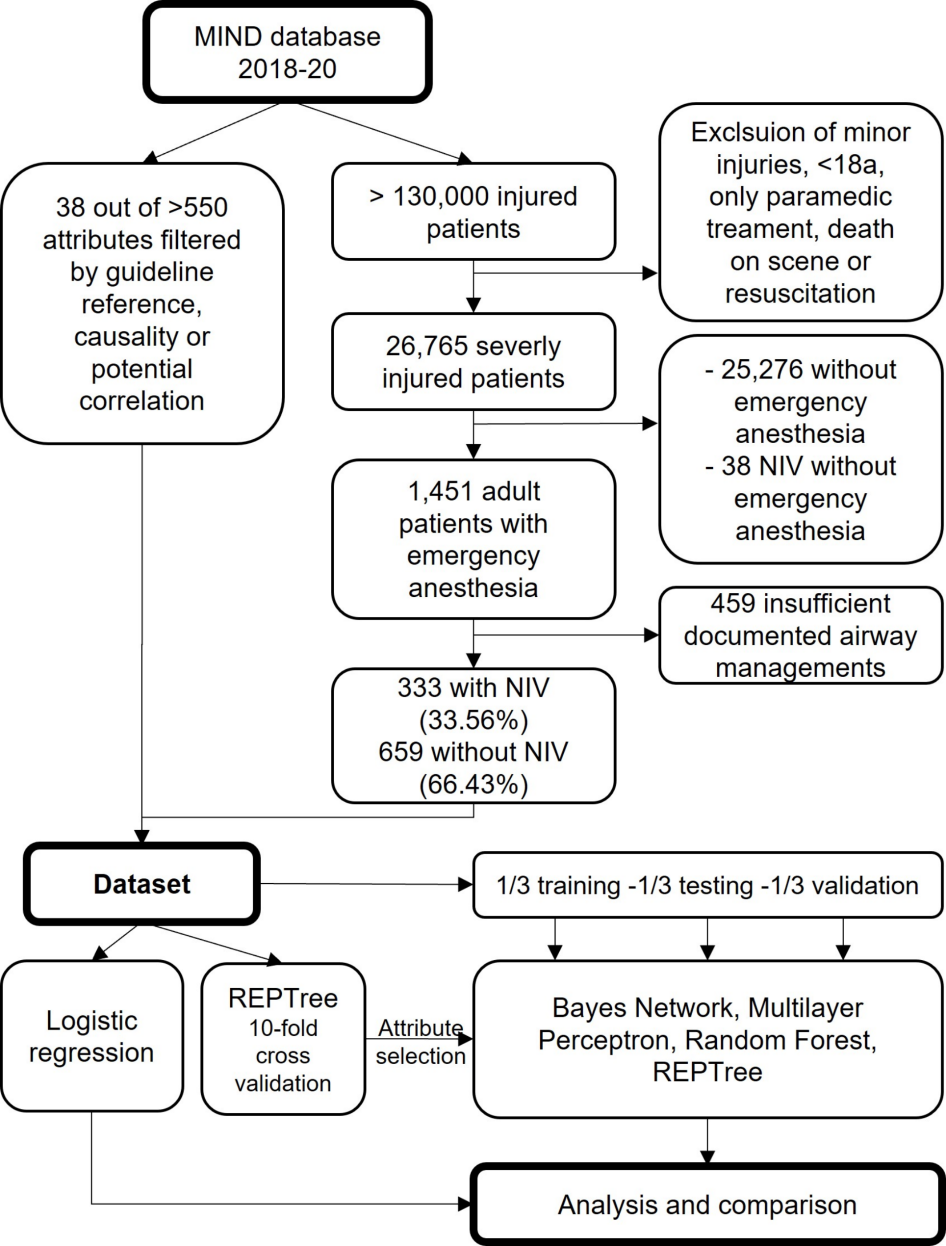
Flow chart with patient selection, dataset creation and analysis. Caption: NIV = non-invasive ventilation, MIND = minimal emergency dataset, REPTree = reduced error pruning tree

This study is a retrospective registry investigation of adult trauma patients, in which prehospital records from 2018 to 2020 were analyzed. The design and the methodology of the study were based on the Transparent Reporting of a Multivariable Prediction Model for individual Prognosis or Diagnosis (TRIPOD) statement [15]. Patient selection, dataset creation and analysis are shown in Fig. 1.

### Settings and population

The German emergency medical service is a paramedic- and emergency physician-based system. Grounded or helicopter emergency physicians are dispatched simultaneously with or requested by a paramedic crew, if certain suspected diagnoses are likely or if pharmacological therapies or invasive techniques, such as airway management, are needed. In general, for the presented study, NIV and airway management in trauma patients were performed only by emergency physicians. German emergency physicians are primarily from the fields of anesthesiology, internal medicine, surgery, or general medicine. The specialization can be achieved in parallel with main medical specialist training after two years of clinical practice, which must include at least a 6-month rotation in the accident and emergency department or intensive care unit. The study region was the south-western German state of Baden-Wuerttemberg (population 11.1 million in 2020, 35,751 km², capital Stuttgart). Statewide, the emergency physicians documented their interventions digitally in a nationwide emergency data set called MIND (minimal emergency dataset). This dataset has already been used for research involving machine learning [10, 16]. Briefly, the analyzable parts of the MIND dataset are divided into subcategories according to the Advanced Trauma Life Support (ABCDE) algorithm. These include the vital signs at first contact and upon hospital admission, suspected injury pattern (not compatible with international trauma scores like the Injury Severity Score (ISS)), suspected diagnosis, auscultation patterns, pharmaceutical therapy and medical interventions (without timing and dosing). Unfortunately, the free text anamnesis and medical history (including a vital sign diagram) are not transferred to the central server for quality assurance due to data protection regulations and therefore cannot be analyzed. The MIND dataset can be linked to the German Trauma Registry and the German Resuscitation Registry [16].

From the MIND-database (2018–2020), adult multiply injured patients were selected who were primarily treated by an emergency physician and required emergency anesthesia at the scene (Fig. 1). Datasets containing prehospital cardiac arrest at any time up to hospital admission were excluded, as the study was on preoxygenation before emergency anesthesia and no information was available about the time of onset of the cardiac arrest relative to invasive airway management. A further inclusion criterion was a complete documentation of airway management to avoid cases where NIV might have been performed but not recorded. Patients were divided into the classes “NIV” and “No NIV”, based on the documented exclusive use of non-invasive ventilation. Generally, prehospital NIV is performed not as a high flow therapy but as a continuous positive airway pressure with or without respiratory support.

### Attribute selection and data preprocessing

Overall, the MIND includes more than 550 attributes, which are mainly dichotomous (e.g., severe head injury: yes or no), with the exception of interval scales like vital signs or nominal scoring systems like the Glasgow Coma Scale (GCS). So far, unlike in internal medicine patients, no definite relationships to NIV in trauma care are known [2]. Therefore, the idea was to consider the NIV as one component of the overall process of emergency anesthesia with regard to guidelines for airway management and trauma care [1, 2]. The national guidelines consider NIV as a preoxygenation method in patients with mild or moderate face or head injuries, the possibility of NIV mask application, low risk of aspiration, patient compliance or preserved consciousness [1, 2]. From the MIND database, attributes representing respiratory distress (auscultation, oxygen saturation, respiratory rate), consciousness (pain level, GCS), the suspected injury pattern by body part (classified as none, mild, moderate, severe or deadly by the attending emergency physician), and aspiration were extracted. Further attributes like vital signs at the first contact (heart rate, systolic blood pressure), pain level, shock index, age and the pre-emergency status (PES, a prehospitally adapted physical status classification of the American Society of Anesthesiologists (ASA)) were also considered as potential attributes. Next, data was screened for potential bias, rarity (leading to the exclusion of the attribute), or statistically significant differences (potential model integration). As most attributes were dichotomous or nominal, imputation of missing data was not considered. Furthermore, documentation of some items, such as PES, was not mandatory, which can lower the attribute´s weighting in a model. To generate a model with only a minimum number of attributes, we first used the attributes occurring in a decision tree model. REPTree, a fast decision tree learner based on the C4.5 algorithm, was used as it already includes a reduced error pruning with backfitting. Its basic method of calculation is the information gain procedure [17]. The goal was to create a tree with a minimum size and the fewest possible attributes to avoid overfitting. Calculation was performed on the entire dataset with ten-fold cross-validation using the machine learning software WEKA (Waikato Environment for Knowledge Analysis, version 3.8.4, University of Waikato, New Zealand, repeated ten-times with a different random number seed). Additionally, the NIV usage was analyzed using logistic regression on the entire dataset (XLSTAT, Lumivero, Denver, CO, USA) and tested with a ten-fold cross-validation in WEKA.

### Machine learning analysis and group comparisons

For supervised machine learning, the attributes used in the first REPTree model were applied to a Random Forest, a multilayer perceptron, Bayes network and a second REPTree model, but this time on the dataset divided into training, testing and internal validation. All these algorithms can handle missing data.

Random Forest (RF) uses multiple random decision trees (in this study *n* = 50) in an ensembled learning method called bagging. The majority classification selected by most trees represents the classification (majority voting). This approach allows RF to address the limitation of overfitting of a single decision tree [18]. The second applied algorithm was a multilayer perceptron model (MLP). MLP is an artificial neural network consisting of an input layer (representing the number of attributes), hidden layers and an output layer (providing the classification). Between each node, there is a weighted connection, calculated as the weighted sum of its inputs through a sigmoid function. During learning, these weights are adjusted by the backpropagation algorithm. The number of hidden layers and their number of nodes is determined experimentally. In this study, we used two hidden layers with three nodes each (details on the calculation are provided in the supplement) [18]. The basic assumption of naïve Bayes is the independence of all attributes. However, in real-world data, conditional dependencies (e.g., state of consciousness and GCS) are often present. The Bayes network (BN) handles this limitation of naïve Bayes with the help of a directed acyclic graph. This graph is constructed from a predefined maximum number of parental nodes that calculate one new child node. Multiple child nodes, within the defined limit, can act as parental nodes for subsequent child nodes, ultimately leading to the root node of the class prediction. For the probability calculation of each node, the Bayesian method of comparing conditional probabilities is applied, linking child and parental nodes via the joint probability function. So, conditional dependencies can be associated. As a network creator, the WEKA`s simple estimator was used to find the conditional probability tables, while the K2 search algorithm was employed to determine the network structure. Currently, no universally accepted technique exists for constructing a network with regard to the maximum number of parental nodes. For this study, we chose a maximum of three parental nodes in K2, based on the number of attributes selected by the decision tree model [19]. Additional information about the settings for each algorithm is provided in the supplement.

### Training, testing, validation, model performance and group comparisons

Initially, the entire dataset was split into two subsets: a training and testing set (66%) and an internal validation set (33%). The training-testing set was further divided in half, and this procedure was repeated ten times with a different random number seed, based on the original training-testing-set. Consequently, the resulting models differed slightly and were tested ten times on the validation set. The general performance criteria included overall correctness, kappa value, the area under the receiver operating characteristic curve (AUC-ROC), sensitivity (NIV), specificity (No NIV), positive and negative predictive value (PPV, NPV). Additionally, the precision-recall-area (PRC-area) was calculated for sensitivity and PPV, as well as specificity and NPV [18]. To evaluate the quality of the two-class classification for datasets of different sizes, the Matthews correlation coefficient was used (MCC, range − 1 total disagreement, 0 random prediction to + 1 perfect prediction) [20]. The lowest overall error rate was automatically chosen for the cost-benefit calculation for all algorithms. The performance across all ten runs of the test and validation set was extracted from WEKA and then averaged in Microsoft Excel 2021. Differences of the performance criteria were tested for significance using a paired t-test. Testing was performed between the training and validation set for each algorithm and also between the algorithms within the validation set.

Statistical analyses including logistic regression, were conducted using Microsoft Excel 2021 with the XLSTAT extension (Microsoft Corporation, Redmond, WA, USA and Lumivero, Denver, CO, USA). All results of group comparisons are presented as means with 95% confidence intervals (95% CI) or medians with interquartile range (IQR), as appropriate. A *p*-value of < 0.05 was considered statistically significant.

## Results

### Dataset and descriptive statistics

The database included over 130,000 datasets of injured patients. After excluding cases with minor injuries, patients under 18 years, treatment by paramedic only, resuscitation, or death on scene, 26,765 patients with multiple injuries and emergency physician treatment remained (including 38 cases with non-invasive ventilation only). Among these 1,451 cases of emergency anesthesia were identified based on documented airway management, the usage of muscle relaxants, or invasive mechanical ventilation documented upon hospital admission. 459 datasets with insufficient documentation of airway management had to be excluded. 992 datasets remained for analysis. Overall missing data were distributed as follows: PES *n* = 328 (33%), oxygen saturation *n* = 9 (1%), pain level *n* = 85 (9%), shock index *n* = 3 (0.3%), heart rate *n* = 3 (0.3%), respiratory rate *n* = 32 (3.3%). The 992 datasets consisted of 333 NIV utilizations (class “NIV”, 33.56%, 75% male patients) and 659 with conventional preoxygenation (bag valve mask or oxygen mask, class “No NIV”, 66.44%, 74% male patients). The male-to-female ratio was not significantly different between the groups (*p* = 0.34) and therefore not considered for prediction. Patients in the class “NIV” were generally younger (mean age 51.8 years (95% CI 49.7–54) vs. 57.2 years (95% CI 54.8–59.8), *p* < 0.01). Aspiration and/or hemoptysis was documented in 0.01% in both classes and therefore not included in further analyses (*p* = 0.65). GCS had a median of 15 in both groups (“NIV” 15 (13 to 15), “No NIV” 15 (14 to 15), *p* = 0.08). The class “NIV” more frequently presented with dyspnea or cyanosis (66% vs. 23%, *p* < 0.01) and bronchial spasm (47% vs. 1%, *p* < 0.01). In contrast, obstruction, gasping, or apnea was more frequent in the class “No NIV” (2% vs. 13%, *p* < 0.01). Regarding the injury pattern, moderate head injury was more prevalent in the “NIV” class (23% vs. 12%, *p* < 0.01). The average oxygen saturation was lower in the “NIV” class (93.4% [95% CI 92.7–94.1] vs. 94.7% [95% CI 93.9–95.5], *p* < 0.01), while the median respiratory rate was higher (15/min [IQR 15–15] vs. 14/min [12–18], *p* < 0.01). No significant differences were observed in heart rate (90/min vs. 89/min) and in systolic blood pressure (138mmHg vs. 137mmHg), with both *p* > 0.5. Further details are provided in Table 1 and supplementary Table 1.

**Table 1:** Clinical attributes for patients with and without NIV

Attribute	Value	NIV (*n* = 333)	No NIV (*n* = 659)	*p*
*Decision tree model and logistic regression*				
**Auscultation**	obstruction/gasping/apnea bronchial spasm rhonchus other normal	2% 47% 4% 1% 47%	13% 1% 7% 4% 76%	< 0.01* < 0.01* 0.1 < 0.01* < 0.01*
**Head injury**	none/mild moderate/severe	24%/6% 23%/47%	38%/4% 12%/45%	< 0.01*/0.2 < 0.01*/0.5
**Age**	years	52 [50–54]	57 [55–60]	< 0.01*
**Heart rate**	/min	90.1 [87.9–92.2]	89.2 [87.1–91.4]	0.5
**Respiratory rate**	/min	15 [15 to 15]	14 [12 to 18]	< 0.01*
**Oxygen saturation**	%	93.4 [92.7–94.1]	94.7 [93.9–95.5]	< 0.01*
*Logistic regression only*				
**Face injury**	none/mild moderate/severe	76%/7% 13%/4%	74%/5% 11%/ 9%	0.6/0.3 0.4/<0.01*
**Cervical spine injury**	none/mild moderate/severe	94%/1% 4%/1%	87%/3% 5%/4%	< 0.01*/0.1 0.5/<0.01*
**Thoracic/lumbar spine injury**	none/mild moderate/severe	92%/1% 3%/4%	88%/2% 6%/4%	0.04*/0.4 0.03*/0.8
**Thoracic injury**	none/mild moderate/severe	68%/2% 12%/18%	69%/2% 10%/18%	0.7/0.9 0.4/0.8
**Abdominal injury**	none/mild moderate/severe	85%/1% 3%/11%	85%/1% 5%/8%	0.9/0.5 0.2/0.3
**Pelvic injury**	none/mild moderate/severe	84%/2% 3%/12%	83%/2% 6%/10%	0.6/0.8 0.06/0.4
**Injury of the soft parts**	none/mild moderate/severe	90%/5% 4%/1%	88%/3% 4%/5%	0.3/0.2 0.9/<0.01*
**PES**	(1–4)	2 (1 to 2)	2 (1 to 2)	0.02*
**GCS**		15 (13 to 15)	15 (14 to 15)	0.08
**Heart rate**	/min	90.1 [87.9–92.2]	89.2 [87.1–91.4]	0.5
**Systolic blood pressure**	mmHg	138 [135–141]	137 [134–140]	0.6
**Pain level**	0 (no pain) − 10	4 (0 to 7)	5 (2 to 8)	< 0.01*
**Shock index**		0.68 [0.65–0.71]	0.7 [0.67–0.73]	0.3

Abbreviations: PES, pre-emergency status with 1 = healthy to 4 = morbibund; GCS, Glasgow Coma Scale; NIV, non-invasive ventilation; results given as percentage, median with interquartile range in parenthesis (respiratory rate) or mean with standard deviation (others); *statistically significant (*p* < 0.05)

### Logistic regression

Attributes with a statistically significant influence (*p* < 0.05) in favour of NIV were bronchial spasm (odds ratio (OR) 119.75), dyspnea/cyanosis (OR 2.28), moderate and severe head injury (both OR 3.37) and the respiratory rate (OR 1.07). Statistically significant attributes for abdication were obstruction/gasping/apnea (OR 0.29), severe face injury (OR 0.38), mild cervical spine injury (OR 0.13), severe pelvis injury (OR 0.04), moderate thoracic/lumbar spine injury (OR 0.3) and the pain level (OR 0.89). The sensitivity of the logistic regression model was 0.56 (95% CI 0.55–0.56), PPV 0.82 (95% CI 0.81–0.82), specificity 0.95 (0.94–0.95) and NPV 0.8 (95% CI 0.8–0.81). It yielded an AUC-ROC of 0.84 (95% CI 0.84–0.84) and a PRC-area of 0.78 for the “NIV” class (95% CI 0.77–0.78) and 0.87 for the “No NIV” class (95% CI 0.87–0.87). The logistic regression model was statistically significant (chi-squared test, *p* < 0.01*, for further details refer to Table 2 and supplementary Table 10).

**Table 2:** Results of the logistic regression analysis (dependent variable NIV usage)

	Coefficient B	Standard error	z	*p*	Odds Ratio	95% conf. interval
**Constant**	2.86	1.98	1.44	0.15		
**Rhonchus**	-0.54	0.39	1.4	0.16	0.58	0.27–1.24
**Auscultation other**	-1.48	0.79	1.86	0.06	0.23	0.05–1.08
**Obstruction/gasping/apnea**	-1.24	0.46	2.68	< 0.01*	0.29	0.12–0.72
**Bronchial spasm**	4.79	0.64	7.42	< 0.01*	119.75	33.85–423.57
**Dyspnea/cyanosis**	0.82	0.23	3.57	< 0.01*	2.28	1.45–3.58
**Head mild**	0.46	0.58	0.8	0.42	1.59	0.51–4.91
**Head moderate**	1.22	0.33	3.7	< 0.01*	3.37	1.77–6.42
**Head severe**	1.21	0.26	4.64	< 0.01*	3.37	2.01–5.62
**Face mild**	0.68	0.41	1.68	0.09	1.98	0.89–4.38
**Face moderate**	0.17	0.3	0.57	0.57	1.18	0.66–2.12
**Face severe**	-0.96	0.47	2.01	0.04*	0.38	0.15–0.98
**Cervical spine mild**	-2.05	0.92	2.24	0.02*	0.13	0.02–0.78
**Cervical spine moderate**	-0.71	0.56	1.29	0.20	0.49	0.16–1.45
**Cervical spine severe**	-0.98	1.05	0.93	0.35	0.38	0.05–2.94
**Thoracic/lumbar spine mild**	0.04	0.93	0.04	0.96	1.04	0.17–6.42
**Thoracic/lumbar spine moderate**	-1.21	0.61	1.97	0.05*	0.3	0.09–0.99
**Thoracic/lumbar spine severe**	-0.5	0.6	0.84	0.40	0.6	0.19–1.96
**Thorax mild**	0.06	0.63	0.09	0.93	1.06	0.31–3.65
**Thorax moderate**	0.14	0.31	0.45	0.65	1.15	0.63–2.1
**Thorax severe**	0.24	0.3	0.79	0.43	1.27	0.7–2.3
**Abdomen mild**	0.6	0.87	0.68	0.49	1.82	0.33–10.05
**Abdomen moderate**	0.19	0.51	0.37	0.71	1.21	0.45–3.27
**Abdomen severe**	0.37	0.35	1.05	0.30	1.44	0.73–2.87
**Pelvis mild**	0.84	0.79	1.07	0.29	2.32	0.49–10.9
**Pelvis moderate**	-0.72	0.59	1.23	0.22	0.49	0.15–1.53
**Pelvis severe**	0.68	0.33	2.04	0.04*	1.98	1.03–3.8
**Soft parts mild**	0.08	0.52	0.16	0.87	1.09	0.39–3.04
**Soft parts moderate**	-0.49	0.54	0.91	0.36	0.61	0.21–1.76
**Soft parts severe**	-0.58	0.68	0.85	0.4	0.56	0.15–2.14
**Thoracic drainage**	-0.36	0.35	1.02	0.31	0.7	0.35–1.39
**PES 2**	-0.01	0.24	0.03	0.97	0.99	0.62–1.6
**PES 3**	0.69	0.38	1.82	0.07	1.99	0.95–4.16
**PES 4**	0.2	0.87	0.23	0.81	1.22	0.22–6.71
**Age**	-0.02	0.01	3.11	< 0.01*	0.98	0.97–0.99
**GCS**	-0.01	0.02	0.64	0.52	0.99	0.94–1.03
**Heart rate**	0.01	0.01	0.86	0.40	1.01	0.99–1.03
**Systolic blood pressure**	-0.01	0.01	1.35	0.18	0.99	0.98–1
**Respiratory rate**	0.07	0.03	2.55	0.01*	1.07	1.02–1.13
**Oxygen saturation**	-0.03	0.01	1.9	0.06	0.97	0.95–1
**Pain level**	-0.11	0.04	3.2	< 0.01*	0.89	0.83–0.96
**Shock index**	-1.96	1.18	1.67	0.10	0.14	0.01–1.41

Logistic regression analysis shows that the model as a whole is significant (chi-squared test (degrees of freedom = 41) = 438.26, *p* < 0.001*, -2 Log-Likelihood 663.01, Cox & Snell R² 0.4, Nagelkerke R² 0.55, McFadden’s R² 0.4), *statistically significant (*p* < 0.05) in a Z-test. The reference categories were normal auscultation, no injury of the certain body part and PES 1. Abbreviations: PES, pre-emergency status with 1 = healthy to 4 = morbibund; GCS, Glasgow Coma Scale; NIV, non-invasive ventilation

### Attribute selection of the first decision tree model

**Fig. 2:**
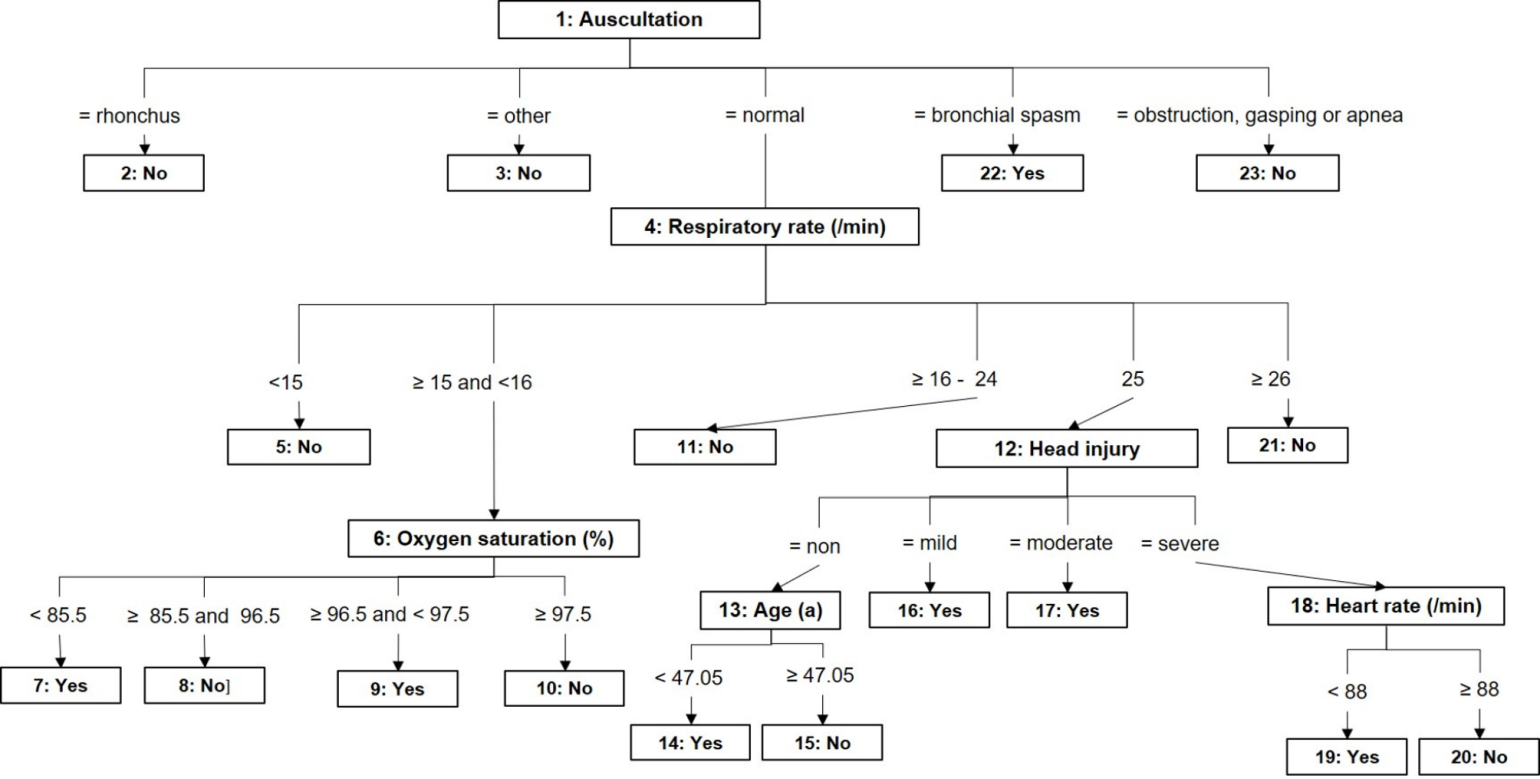
REPTree decision tree. Caption: The structure of this decision tree derives from the available data. Slight variations can lead to significant changes. Although it may reflect to some extend a clinical approach, it must be interpreted more as a model derived from the observed data rather than a definitive clinical rule. For further details on the amount of (in-) correctly classified instances from the training and pruning set refer to the supplement. REPTree = reduced error pruning tree

The decision tree had 23 nodes with 17 leaves and consisted of the six attributes: auscultation, head injury, respiratory rate, oxygen saturation, age and heart rate (Fig. 2). Key elements were NIV usage in bronchial spasm, desaturation, and mild or moderate head injury. It achieved an AUC-ROC of 0.93 (95% CI 0.92–0.93) and a PRC-area of 0.89 for “NIV” (95% CI 0.87–0.90) and 0.94 for “No NIV” (95% CI 0.93–0.94). Particulars about classification results (the amount of (in-) correctly classified instances from the training and pruning set), and a detailed interpretation are given in supplementary Fig. 1.

### Performance of the machine learning algorithms

**Fig. 3:**
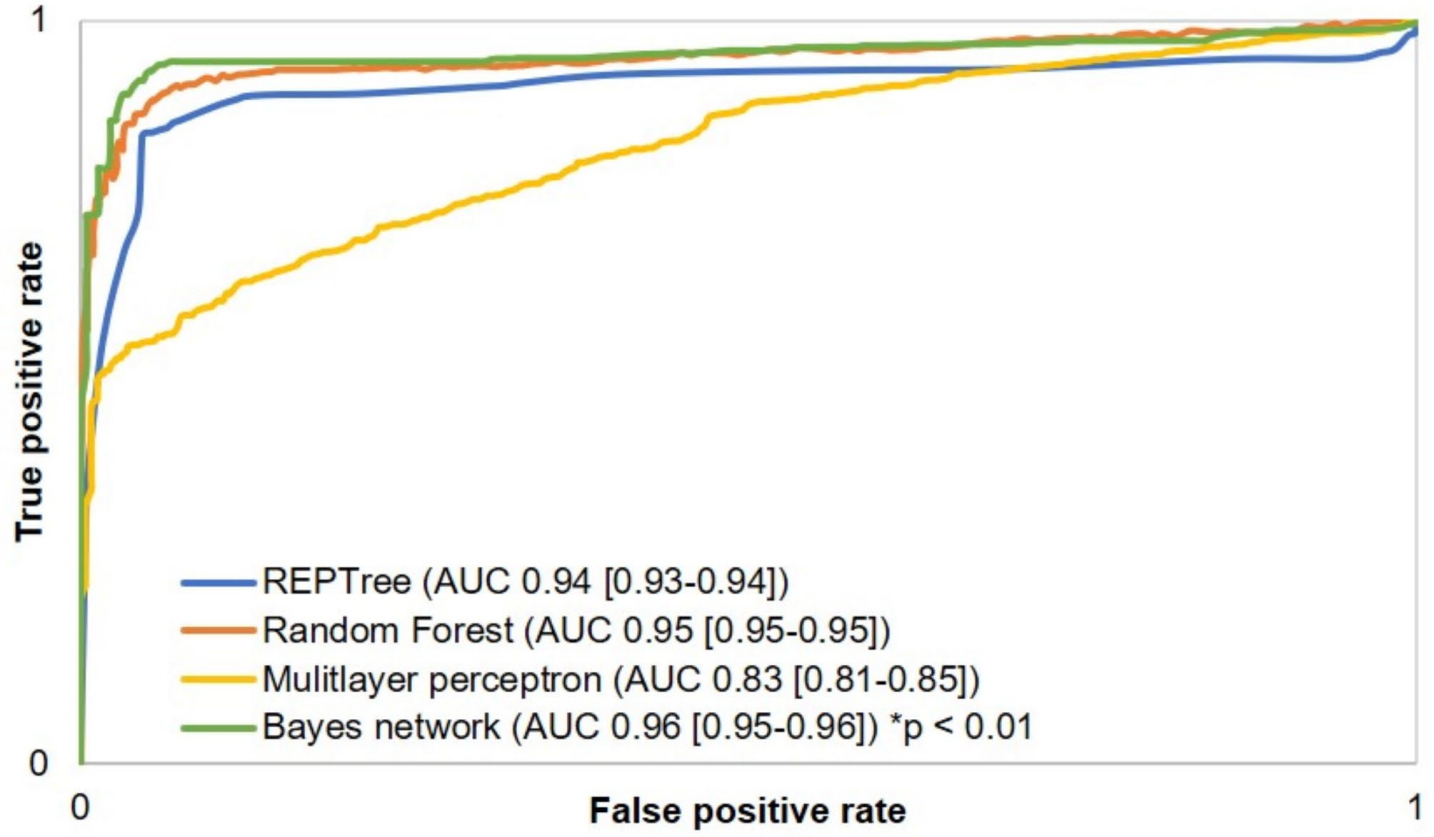
Averaged area under the receiver operating characteristic curve (AUC) of the validation data set for the overall performance of prediction and exclusion of non-invasive ventilation for preoxygenation of the Bayes network, REPTree, Random Forest and Multilayer Perceptron. Caption: Area under the receiver operating characteristic curve (AUC) from the respective algorithm, 95% confidence interval in parenthesis, REPTree = reduced error pruning tree, *p-value for comparison of Bayes network versus all other algorithms

**Fig. 4:**
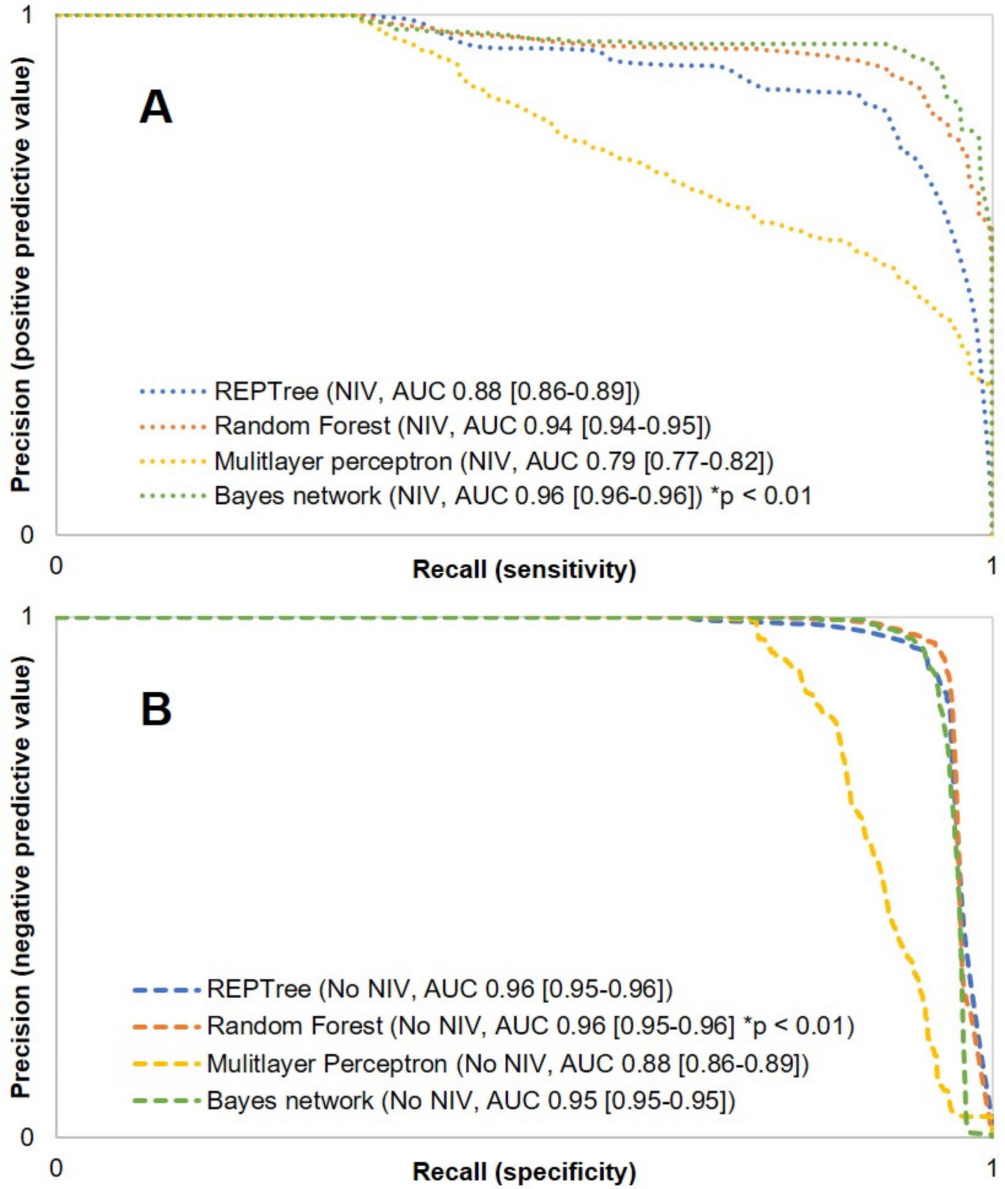
Averaged precision-recall-curves (PRC) of the validation set for the prediction (**A**) and exclusion of non-invasive ventilation (**B**) of non-invasive ventilation for preoxygenation of the Bayes network, REPTree, multilayer perceptron and Random Forest (B). Caption: Area under the curve (AUC) from the respective algorithm, 95% confidence interval in parenthesis, REPTree = reduced error pruning tree, MLP = multilayer perceptron, *p-value for comparison of Bayes network (**A**) or rather (**B**) Random Forest versus all other algorithms

Based on the attributes occurring in the first decision tree model, the training, testing and validation of the four models were performed. The training and validation results of RF, REPTree, BN and MLP are given in Tables 3 and 4. Except for the MLP, all other algorithms yielded comparable results. Summarized, on the validation set, the Bayes network had the highest rate of 94.76% total correctness (95% CI 94.66–94.86), of 0.91 sensitivity (95% CI 0.91–0.91), of 0.97 specificity (95% CI 0.97–0.97), of 0.93 PPV (95% CI 0.93–0.93) and of 0.96 NPV (95% CI 0.96–0.96). It also yielded the highest AUC-ROC of 0.96 (95% CI 0.95–0.96) and PRC-area for NIV usage (95% CI 0.96, 0.96–0.96). RF performed marginally better in the PRC-area for the exclusion of NIV (0.96 [95% CI 0.96–0.96] compared to BN (0.95 [95% CI 0.95–0.95]). From testing to validation, only the MLP showed improved results on the validation set, whereas the other tree algorithms predicted robustly. AUC-ROCs and PRC-curves are shown in Figs. 3 and 4.

**Table 3:** Direct comparison and performance evaluation of the other four algorithms on the testing and validation set

	*REPTree*			*Bayes network*			
	*Testing*	*Validation*	*p*	*Testing*	*Validation*	*p*	
Correctness (%)	89.64 [87.93–91.34]	91.36 [90.59–92.14]	0.07	94.53 [93.61–95.44]	94.76 [94.66–94.86]	0.34	
Kappa	0.76 [0.73–0.8]	0.8 [0.78–0.82]	0.1	0.88 [0.86–0.9]	0.88 [0.88–0.88]	0.67	
MCC	0.77 [0.73–0.81]	0.8 [0.78–0.82]	0.11	0.88 [0.86–0.9]	0.88 [0.88–0.88]	0.7	
Threshold	0.77 [0.73–0.81]	0.71 [0.62–0.81]	0.29	0.54 [0.51–0.56]	0.56 [0.55–0.57]	0.09	
AUC-ROC	0.91 [0.9–0.93]	0.94 [0.93–0.94]	0.04*	0.96 [0.95–0.96]	0.96 [0.95–0.96]	0.88	
Sensitivity	0.81 [0.78–0.85]	0.84 [0.83–0.86]	0.13	0.91 [0.9–0.93]	0.91[0.91–0.91]	0.4	
PPV	0.87 [0.84–0.9]	0.88 [0.87–0.9]	0.48	0.92 [0.89–0.95]	0.93 [0.93–0.93]	0.49	
PRC-area	0.86 [0.83–0.9]	0.88 [0.86–0.89]	0.44	0.95 [0.95–0.96]	0.96 [0.96–0.96]	0.59	
Specificity	0.94 [0.92–0.96]	0.95 [0.94–0.95]	0.34	0.96 [0.95–0.98]	0.97 [0.97–0.97]	0.4	
NPV	0.91 [0.89–0.92]	0.93 [0.92–0.93]	0.04*	0.96 [0.95–0.97]	0.96 [0.96–0.96]	0.74	
PRC-area	0.93 [0.91–0.94]	0.96 [0.95–0.96]	< 0.01*	0.96 [0.95–0.96]	0.95 [0.95–0.95]	0.16	
	**Random Forest**			**Multilayer perceptron**			
	*Testing*	*Validation*	*p*	*Testing*	*Validation*	*p*	
Correctness (%)	93.53 [92.79–94.28]	93.15 [92.87–93.43]	0.32	79.52 [78.06–80.98]	83.06 [81.35–84.77]	< 0.01*	
Kappa	0.85 [0.84–0.87]	0.84 [0.84–0.85]	0.24	0.51 [0.48–0.55]	0.58[0.53–0.63]	0.03*	
MCC	0.85 [0.84–0.87]	0.84 [0.84–0.85]	0.17	0.53 [0.5–0.57]	0.6 [0.56–0.64]	0.02*	
Threshold	0.51 [0.5–0.53]	0.51 [0.5–0.52]	0.69	0.56 [0.51–0.61]	0.52 [0.5–0.54]	0.17	
AUC-ROC	0.96 [0.95–0.97]	0.95 [0.95–0.95]	0.02*	0.81 [0.79–0.83]	0.83 [0.81–0.85]	0.11	
Sensitivity	0.86 [0.84–0.88]	0.88 [0.87–0.88]	0.22	0.58 [0.51–0.65]	0.61 [0.52–0.7]	0.59	
PPV	0.94 [0.93–0.95]	0.91 [0.9–0.92]	< 0.01*	0.75 [0.66–0.84]	0.85 [0.8–0.9]	0.06	
PRC-area	0.96 [0.95–0.96]	0.94 [0.94–0.95]	0.01*	0.76 [0.74–0.78]	0.79 [0.77–0.82]	0.06	
Specificity	0.97 [0.97–0.98]	0.96 [0.95–0.96]	< 0.01*	0.9 [0.86–0.95]	0.95 [0.93–0.97]	0.06	
NPV	0.94 [0.92–0.95]	0.94 [0.94–0.94]	0.51	0.81 [0.78–0.84]	0.85 [0.81–0.88]	0.14	
PRC-area	0.96 [0.95–0.97]	0.96 [0.95–0.96]	0.39	0.86 [0.83–0.9]	0.88 [0.86–0.89]	0.46	

95% confidence interval given in parentheses, abbreviations: AUC-ROC, area under the receiver operating characteristic curve; PPV = positive predictive value; NPV = negative predictive value; MCC, Matthews correlation coefficient; PRC-area, precision-recall-area (given for the prediction and exclusion of non-invasive ventilation); REPTree = reduced error pruning tree, *statistically significant value (*p* < 0.05)

**Table 4:** Performance evaluation of the other four models on the validation set

	*Bayes Network*	*Random Forest*	*REPTree*	*Multilayer perceptron*
Correctness (%)	94.76 [94.66–94.86]	< 0.01*	< 0.01*	< 0.01*
Kappa	0.88 [0.88–0.88]	< 0.01*	< 0.01*	< 0.01*
MCC	0.88 [0.88–0.88]	< 0.01*	< 0.01*	< 0.01*
AUC-ROC	0.96 [0.95–0.96]	< 0.01*	< 0.01*	< 0.01*
Sensitivity	0.91 [0.91–0.91]	< 0.01*	< 0.01*	< 0.01*
PPV	0.93 [0.93–0.93]	< 0.01*	< 0.01*	< 0.01*
PRC-area	0.96 [0.96–0.96]	< 0.01*	< 0.01*	< 0.01*
Specificity	0.97 [0.97–0.97]	< 0.01*	< 0.01*	< 0.01*
NPV	0.96 [0.96–0.96]	< 0.01*	< 0.01*	< 0.01*
PRC-area	< 0.01*	0.96 [0.95–0.96]	< 0.01*	< 0.01*

95% confidence interval given in parentheses, abbreviations: AUC-ROC, area under the receiver operating characteristic curve; PPV = positive predictive value; NPV = negative predictive value; MCC, Matthews correlation coefficient; PRC-area, precision-recall-area (given for the prediction and exclusion of non-invasive ventilation); REPTree = reduced error pruning tree, *statistically significant value (*p* < 0.05)

## Discussion

The aim of the study was to characterize injured patients treated with NIV prior to prehospital emergency anesthesia with the help of machine learning. NIV was applied in about one-third of all emergency anesthesia in severely injured patients. In the decision tree, these patients were characterized by mainly mild to moderate head injuries and the presence of dyspnea, cyanosis, or bronchial spasm. Additionally, severe pelvic and facial injuries, or mild spinal injuries, as well as a high pain level, were associated with a lower likelihood of NIV usage in the logistic regression analysis. The decision tree model, Random Forest and Bayesian network all demonstrated an excellent class discrimination, with the differences unlikely to be clinically relevant. In contrast, the performance of MLP and logistic regression was inferior to the other machine learning methods applied in this study. Machine learning models for NIV have previously been developed for applications such as respiratory deterioration, extubation failure, or duration of respiratory support [21–24]. To the best of our knowledge, this investigation is the first to use machine learning for an analysis of NIV in a prehospital trauma setting [25–29]. However, several factors need to be considered when interpreting and drawing conclusions from the results.

### Guideline adherence and clinical implications

The evidence regarding the utilization of NIV in general trauma care is limited. The combined guideline of the European Respiratory Society and American Thoracic Society suggest that NIV usage in chest trauma can decrease the need for intubation, the incidence of nosocomial pneumonia, length of stay in the intensive care unit, and mortality. However, the optimal begin and duration remain unclear [6]. Fong et al. demonstrated in their meta-analysis of preoxygenation strategies that patients with NIV experienced less desaturation and fewer intubation-related complications, but no significant difference in mortality [30]. Unfortunately, trauma patients were not analyzed separately in the meta-analysis or the enrolled studies [31–34]. Clinically, not every patient is suitable for non-invasive ventilation. National and international guidelines recommend avoiding NIV in severe face injury, anatomical or injury-related impossibility of NIV mask application, high risk of aspiration, or patient incompliance [2, 5, 6]. All these conditions are particularly common in trauma patients, making non-invasive preoxygenation challenging and necessitating highly skilled emergency physicians [35]. The documented low rates of aspiration and of reduced consciousness in this study suggest that emergency physicians largely adhered to these guidelines when selecting a preoxygenation method. Taking the decision tree results into account, trauma patients without these contraindications could be suitable candidates for alternative preoxygenation approaches. Furthermore, in cases where invasive airway protection is unavoidable and predictors of a potentially difficult airway are present, NIV could be considered for preoxygenation on a case-by-case basis. However, this approach is not fully reflected in the available data structure, as the MIND database lacked information on free text entries, trends in scores, body weight and size, airway anatomy and additional vital signs. Consequently, this study cannot draw conclusions about the effectiveness of NIV, its impact on patient outcome, or potential adverse effects, as in-hospital clinical course data were not available. Despite these limitations, this study provides valuable insights into the prevalence and determinants of NIV utilization as preoxygenation technique in trauma scenarios.

Regarding the attributes in the MIND, it should be emphasized that attributes like respiratory rate were likely often estimated rather than accurately measured during routine prehospital work. It should be investigated in a controlled environment, if such estimations might have really influenced the likelihood of selecting a specific preoxygenation technique. Furthermore, the mathematical algorithm behind the splitting procedure of the decision tree loosely constructed connected decisions, such as heart rate and head injury. This phenomenon can be attributed not only to the limitations of the MIND dataset or the inherent instability of decision trees - where slight variations in data can lead to significant changes in the model - but also to the relatively small sample size. Therefore, it is crucial to interpret the decision tree as a model derived from the observed data rather than a definitive clinical rule. Nonetheless, it may reflect the clinical approach used to identify patients suitable for NIV. Factors such as auscultation findings, oxygen saturation (after oxygen delivery), normo- or tachypnea and injury pattern were likely instrumental in clinical decision-making. To some degree, these factors align with the guideline recommendations, although they do not fully account for the level of consciousness and aspiration risk [1, 2]. It should also be noted that while some differences in vital signs between the groups were statistically significant, they were often clinically not meaningful. This underscores the value of incorporating machine learning approaches, which excel at handling large and complex datasets with a focus on improving prediction accuracy and generalization.

### Database and attribute selection

In general, models with excellent class discrimination, as presented here, are at risk of overfitting [18]. However, several measures were implemented to mitigate this issue. First, from the authors’ point of view, none of the attributes were exclusively linked to one class in advance. For example, even a low GCS combined with low oxygen saturation could justify the use of NIV for preoxygenation. With NIV applied in 333 emergency anesthesia and only 38 (10.2%) additional cases without any documented airway management excluded from the analyses, there is no evidence suggesting a general failure of this technique or its use as a preventive measure against invasive airway management. Second, the decision tree model demonstrated that patient selection was mainly based on universal attributes like auscultation, respiratory rate, oxygen saturation and head injury. By these attributes alone, an excellent class discrimination could be achieved. Also, the stability of these attributes was confirmed across all testing and validation procedures. Additionally, the use of NIV may reflect a more advanced approach to emergency anesthesia, as it was associated with more frequent videolaryngoscopy use, fewer documented difficult airways, and often performed by anesthesiologist (see supplementary Table 1) [36, 37]. Besides, this could also suggest a potential lack of training among non-anesthesiologist emergency physicians.

In internal medicine, non-invasive ventilation strategies are frequently applied in respiratory failure, particularly in obstructive respiratory failure [5, 6]. In this study, the decision tree (and the logistic regression) identified bronchial spasm as the primary attribute. In trauma cases, bronchospasm can result from thoracic trauma with lung contusion [28, 38, 39]. Conversely, rhonchus may indicate aspiration, a contraindication for NIV. Yet, documented aspiration was not more frequent in the NIV class, aligning with the findings of Gibbs et al. [8]. Moreover, data on pre-existing conditions like chronic obstructive pulmonary disease were not available in the MIND. Also, intrathoracic and intraabdominal injuries might not be reliably excluded through external clinical examinations due to the prehospital lack of radiographic diagnostics. This limitation could explain the similar injury patterns observed for these injuries in patients with and without NIV.

### Influence on further model development

Although the results of the model comparison, with their excellent class discrimination, are promising, they represent only a first step in model development [40]. For a future machine learning-based support system in prehospital airway management, the development process will need to address several complex factors, including the correct indication, performance, patient-specific effectiveness, and clinical outcome of the chosen methods [41]. Nevertheless, the results of this study suggest that modeling the human decision-making process is feasible and provide a first step toward identifying the most effective algorithms. With the exception of the MLP and logistic regression, all algorithms performed robustly and consistently. With regard to a future model, BN and RF were the most promising algorithms. BNs offer advantages such as network visualization and handling attribute dependency. They are also more adaptable, making them potentially extensible to other domains, such as internal emergency patients. Yet, BN require a careful attribute selection to avoid cyclic relationships of the discriminable attributes [19]. The probability of such cycles rises with the maximum number of parental nodes. However, in this study, no such cycles were identified in the network. Also, BNs tend to be more stable with regard to change in data structure compared to a single decision tree model. By aggregating results from multiple decision trees (bagging procedure), Random Forest, on the other hand, had a more robust prediction compared with a single decision tree. This robustness enhances its reliability in varied datasets. With regard to the results of MLP, a deep learning approach applied to a larger dataset could offer an alternative for improving results [17]. The insufficient results of the logistic regression in this context likely could stem not only from the non-linear relationships within the data (e.g., respiratory or heart rate), but also from multicollinearity (e.g., respiratory rate, auscultation findings, oxygen saturation) and from outliners. While logistic regression struggles with such complexities and multicollinearity, decision trees excel due to their ability to separate data non-linearly [18, 42]. This is evident in the weighting of severe head injury in the decision tree model (Fig. 2), which shows dependencies with attributes like auscultation, respiratory rate, and heart rate. These relationships are expressed probabilistically in the tree model, in contrast to the odds ratios produced by logistic regression.

### Limitations

Mainly, limitations of this study stem from the data structure. The MIND only contains data on vital signs recorded at the first contact and upon hospital admission. Due to the absence of further medical records, it was not possible to assess the effectiveness of the chosen technique or its effect on the clinical outcome. Unfortunately, even an analysis of the oxygen saturation, blood pressure or end-tidal carbon dioxide on admission would not have been constructive, as key details such as catecholamine dosages or ventilator settings were not recorded. A linkage to trauma scores like the ISS could enhance further international studies. However, this would require modifications of the dataset, including reducing the number of the body regions (from eight to six) and adapting the injury pattern (from a five- to a six-point scale). Furthermore, the filtered dataset used in this study was relatively small for validation through data splitting, so cross-validation was also employed, yielding comparable results (see supplement) [18]. Although the study demonstrated an excellent class discrimination, the algorithms were developed using retrospective data from a state-wide emergency medical service and therefore have to be tested in an independent external cohort. Thus, predictions about stability with regard to noise and overfitting are limited. Interpolation of missing values was not possible because the analyzed parameters were static. Data on defective equipment were also not available. As the results were developed in a physician staffed emergency medical system, they cannot not simply be transferred to paramedic systems [43]. Until future research provides broader insights into the use of NIV in trauma care across different regions worldwide, the application of these results remains confined to retrospective research, such as identifying suitable patients for NIV.

## Conclusion

In this study, we analyzed a cohort of severely injured adults requiring prehospital emergency anesthesia using machine learning methods to identify predictors of non-invasive ventilation as preoxygenation method. Patients receiving NIV more frequently presented with dyspnea, cyanosis, or bronchial spasm and predominantly had the presumptive diagnosis of no up to moderate head injury. We found that NIV was applied in about one third of all prehospital emergency anesthesia in trauma patients. These findings align mostly with current national guidelines [1, 2]. However, the evidence level of NIV in prehospital trauma care, especially during preoxygenation, remains limited. As data on the effectiveness of NIV and its impact on clinical outcome are lacking, further studies with an extended database need to be conducted.

## Electronic supplementary material

Below is the link to the electronic supplementary material.

Supplementary Material 1 - Model Bayes Network

Supplementary Material 2 - Model Multilayer perceptron

Supplementary Material 3 - Model REPTree

Supplementary Material 4 - Model Random Forest

Supplementary Material 5 - Supplemetary data

## Data Availability

Due to data protection, the dataset cannot be published, but research with the database is possible upon request to the Center for Quality Management in Emergency Medical Services Baden-Wuerttemberg (SQR-BW). The supplemented models are free to use.

## Abbreviations

ASA: American society of anesthesiologists
AUC: Area under the curve
BN: Bayes network
GCS: Glasgow coma scale
IQR: interquartile range
ISS: Injury severity score
MCC: Matthews correlation coefficient
MIND: Minimal emergency dataset
MLP: multilayer perceptron model
NIV: Non–invasive ventilation
NPV: Negative predictive value
PES: Pre–emergency status
PPV: Positive predictive value
PRC: Recession–recall–area
REPTree: Reduced error pruning tree
RF: Random forest
ROC: Receiver–operator characteristics
TRIPOD: Transparent reporting of a multivariable prediction model for individual prognosis or diagnosis
WEKA: Waikato environment for knowledge analysis
