# Supplementary material for: Utilization of non-invasive ventilation before prehospital emergency anesthesia in trauma – a cohort analysis with machine learning
Source: Scand J Trauma Resusc Emerg Med. 2025 Mar 3;33:35. doi: 10.1186/s13049-025-01350-1 (PMC11877787; doi:10.1186/s13049-025-01350-1)
Supplement: Supplementary file 5 — Supplementary Material 5 - Supplemetary data [file 13049_2025_1350_MOESM5_ESM.docx]

**Utilization of non-invasive ventilation before prehospital emergency anesthesia in trauma – a cohort analysis with machine learning**

**Supplement information**

| Supplementary Table 1 | Clinical findings and medical treatments for both classes with attributes not selected from the decision tree model | Page 2 |
| --- | --- | --- |
| Supplementary Table 2 | Clinical attributes for patients with and without NIV selected in the decision tree model separated in training, testing and validation set | Page 3 |
| Supplementary Table 3 | Settings of the REPTree algorithm in WEKA | Page 4 |
| Supplementary Table 4 | Settings of the Bayesian network with a maximum of 3 parental nodes together with estimator “simple estimator” and the search algorithm “K2” in WEKA | Page 4 |
| Supplementary Table 5 | Settings of the Random Forest algorithm in WEKA | Page 5 |
| Supplementary Table 6 | Settings of the multilayer perceptron algorithm in WEKA | Page 6 |
| Supplementary Table 7 | Approach to network design of the multilayer perceptron in a cross-validation approach | Page 7 |
| Supplementary Table 8 | Attribute importance of the Random Forest models | Page 7 |
| Supplementary Table 9 | Results of the cross-validation approach of the RepTree, Bayes Network, Random Forest and multilayer perceptron model | Page 8 |
| Supplementary Table 10 | Results of the logistic regression analysis based on the attributes of the decision tree model | Page 9 |
| Supplementary Figure 1 | REPTree decision tree of the attribute selection process with performance results and comment on structure and interpretation | Page 11 |

| **Supplementary Table 1** Clinical findings and medical treatments for both classes without attributes selected in the models | | | | |
| --- | --- | --- | --- | --- |
| **Attribute** | **Value** | **NIV (n = 333)** | **No NIV (n = 659)** | **p** |
| **Supraglottic airway device** |  | 2% | 5% | 0.06 |
| **Endotracheal intubation** |  | 99% | 95% | <0.01* |
| **Videolaryngoscopy** |  | 58% | 13% | < 0.01* |
| **Surgical airway** |  | 0.3% | 0.3% | 0.9 |
| **Cervical collar** |  | 86% | 62% | <0.01* |
| **Thoracic drainage** |  | 14% | 12% | 0.45 |
| **Difficult mask ventilation** |  | 1% | 0% | <0.01* |
| **Difficult airway anatomy** |  | 5% | 4% | 0.4 |
| **> 2 intubation attempts** |  | 0% | 5% | <0.01* |
| **Strong opioid** | (e. g. fentanyl) | 90% | 79% | <0.01* |
| **Moderate opioid** | (e. g. morphine) | 1% | 2% | <0.01* |
| **Benzodiazepines** | (e. g. midazolam) | 86% | 70% | <0.01* |
| **Muscle relaxant** |  | 86% | 83% | 0.3 |
| **Ketamine** |  | 49% | 43% | 0.1 |
| **Catecholamines** |  | 38% | 44% | 0.06 |
| **Propofol** |  | 50% | 50% | 0.9 |
| **Etomidate** |  | 3% | 3% | 0.9 |
| **Thiopental** |  | 6% | 8% | 0.3 |
| **Emergency physician** | anaesthetist | 98.9% | 68.6% | <0.01* |
| **Mode of transport** | air ambulance | 78% | 18% | <0.01* |
| The proportion of anaesthetists for the whole cohort was 78.29%. Abbreviations: PES, pre-emergency status with 1 = healthy to 4 = morbibund; GCS, Glasgow Coma Scale; NIV, non-invasive ventilation; results given as percentage, mean with standard deviation or median with interquartile range in braces; *statistically significant value (p < 0.05) | | | | |

| **Supplementary Table 2** Clinical attributes for patients with and without NIV selected in the decision tree model separated in training, testing and validation set | | | | | | |
| --- | --- | --- | --- | --- | --- | --- |
|  | **Training (n = 331)** | | **Testing (n = 331)** | | **Validation (n = 330)** | |
|  | **NIV** | **No NIV** | **NIV** | **No NIV** | **NIV** | **No NIV** |
|  | 36% | 64% | 32% | 68% | 32 % | **68%** |
| **Auscultation**  obstruction/gasping/apnea  bronchial spasm  rhonchus  other  normal |  | | | | | |
|  | 1% | 7% | 1% | 10% | 1% | 9% |
|  | 16% | 0% | 16% | 1% | 15% | 0% |
|  | 1% | 4% | 3% | 5% | 1% | 5% |
|  | 0% | 2% | 0% | 3% | 0% | 2% |
|  | 19% | 51% | 13% | 48% | 15% | 51% |
| **Head injury**  none  mild  moderate  severe |  | | | | | |
|  | 10% | 24% | 6% | 27% | 8% | 26% |
|  | 1% | 3% | 2% | 3% | 3% | 2% |
|  | 7% | 6% | 8% | 10% | 8% | 8% |
|  | 18% | 31% | 16% | 27% | 14% | 31% |
| **Age** (years) | 52  ± 20.2 | 57.7  ± 21 | 49.6  ± 22.8 | 55.6  ± 18.1 | 53.9  ± 21.5 | 58.1  ± 20.3 |
| **Heart rate** (/min) | 89  ± 19.7 | 88.8  ± 19.4 | 90.5  ± 21.6 | 88.1  ± 21.2 | 90.1  ± 18.7 | 90  ± 18.3 |
| **Respiratory rate** (/min) | 15  [15 to 15] | 15  [12 to 18] | 14  [12 to 18] | 15  [15 to 15] | 15  [13 to 18] | 15  [15 to 15] |
| **Oxygen saturation** (%) | 93  ± 7.0 | 93.8  ± 6.8 | 93.3  ± 6.0 | 94  ± 5.7 | 93.2  ± 8.4 | 92.9  ± 6.1 |
| Abbreviations: NIV, non-invasive ventilation; results given as percentage, median with interquartile range in parenthesis (respiratory rate) or mean with standard deviation (others); *statistically significant value (p < 0.05) | | | | | | |

**Supplementary Table 3** Settings of the REPTree algorithm in WEKA

| **Setting** | **RepTree** |
| --- | --- |
| batch size | 100 |
| initial count | 0.0 |
| minimal proportions of the variance | 0.001 |
| debug | false |
| do not check capabilities | false |
| number of decimal places | 2 |
| max depth | unlimited |
| No pruning | false |
| Number of folds | 3 |
| Spread initial count | false |

**Supplementary Table 4** Settings of the Bayesian network with a maximum of 3 parental nodes together with estimator “simple estimator” and the search algorithm “K2” in WEKA

| **Setting** | **Bayesian network** | **Setting** | **K2** |
| --- | --- | --- | --- |
| batch size | 100 | Initiate as Naïve Bayes | True |
| debug | false | Markov Blanket classifier | False |
| do not check capabilities | false | Maximum of parental nodes | 1/2/3/5 |
| number of decimal places | 2 | Random Order | False |
| ADTree | no | Score Type | Bayes |
| Estimator | Simple Estimator |  |  |

**Supplementary Table 5** Settings of the Random Forest algorithm in WEKA

| **Setting** | **Random Forest** |
| --- | --- |
| batch size | 100 |
| Bag size percentage | 100 |
| Break ties randomly | false |
| Calc out of bag | false |
| Debung | false |
| Do not check capabilities | False |
| max depth | 0 (unlimited) |
| Number decimal places | 2 |
| Number execution slots | 1 |
| Number features | 0 |
| Number iterations | 50 |
| Out of bag complexity statistics | False |
| Store out of bag predicitions | False |

**Supplementary Table 6** Settings of the multilayer perceptron algorithm in WEKA

| **Setting** | **Mulilayer Perceptron** |
| --- | --- |
| GUI | False |
| Autobuild | True |
| Batch size | 100 |
| Debug | false |
| Decay | false |
| Do not check capabilities | False |
| Hidden layers | 3/3 |
| Learning rate | 0.2 |
| Momentum | 0.1 |
| Nominal to binary filter | true |
| Normalize attributes | True |
| Normalize numeric classes | True |
| Number decimal places | 2 |
| Reset | True |
| Training time | 2000 |
| Validation set size | 0 |
| Validation threshold | 20 |

**Supplementary Table 7** Approach to network design of the multilayer perceptron in a cross-validation approach

|  | Hidden layers | | | |
| --- | --- | --- | --- | --- |
|  | **None** | **3** | **3/3** | **4/4** |
| Correctness (%) | 80.27 ± 3.36 | 81.18 ± 3.54 | 81.28 ± 3.58 | 81.18 ± 3.88 |
| MCC | 0.55 ± 0.08 | 0.56 ± 0.09 | 0.58 ± 0.09 | 0.57 ± 0.09 |
| AUC-ROC | 0.82 ± 0.04 | 0.82 ± 0.05 | 0.82 ± 0.04 | 0.82 ± 0.05 |
| Averaged PRC-area | 0.77 | 0.78 | 0.78 | 0.78 |

All tests were not statistically significant in a paired t-test (all p > 0.05). However, as the model with 3/3 hidden layers performed slightly better, it was chosen for further testing. All networks had 13 input layers (attributes with their values), the given number of hidden layers and two output layers (non-invasive ventilation yes or no).

**Supplementary Table 8** Attribute importance of the Random Forest models

|  | **Age** | **Heart rate** | **Respiratory rate** | **Oxygen saturation** | **Head injury** | **Auscultation** |
| --- | --- | --- | --- | --- | --- | --- |
| **Training/Testing** | 0.4  [0.39-0.4] | 0.35  [0.34-0.35] | 0.33  [0.32-0.33] | 0.3  [0.29-0.31] | 0.29  [0.29-0.3] | 0.25  [0.23-0.26] |
| **Cross Validtation** | 0.39  [0.38-0.39] | 0.33  [0.32-0.33] | 0.32  [0.31-0.32] | 0.29  [0.28-0.29] | 0.27  [0.26-0.28] | 0.24  [0.22-0.25] |
| **p-value** | 0.84 | 0.56 | 0.70 | 0.59 | 0.37 | 0.67 |

Auscultation includes obstruction/gasping/apnea, bronchial spasm, rhonchus, other and normal findings. Head injury includes non, mild, moderate and severe injury.

**Supplementary Table 9** Results of the cross-validation approach of the RepTree, Bayes Network, Random Forest and multilayer perceptron model

|  | Bayes Network | Random Forest | REPTree | Multilayer perceptron |
| --- | --- | --- | --- | --- |
| Correctness (%) | 95.35 [95.17-95.53]* | 94.23 [94.11-94.36] | 91.19 [90.73-91.65] | 83.12 [82.23-84.02] |
| Kappa | 0.89 [0.89-0.9]* | 0.87 [0.87-0.87] | 0.8 [0.79-0.81] | 0.59 [0.57-0.62] |
| MCC | 0.9 [0.89-0.9]* | 0.87 [0.87-0.87] | 0.8 [0.79-0.81] | 0.61 [0.59-0.63] |
| Treshold | 0.54 [0.52-0.55*] | 0.51 [0.5-0.51] | 0.53 [0.5-0.57] | 0.51 [0.5-0.52] |
| AUC-ROC | 0.96 [0.96-0.96]* | 0.96 [0.95-0.96] | 0.93 [0.92-0.93] | 0.84 [0.82-0.85] |
| Sensitivity | 0.95 [0.95-0.95]* | 0.88 [0.88-0.89] | 0.9 [0.89-0.91] | 0.61 [0.58-0.63] |
| PPV | 0.91 [0.9-0.91]* | 0.94 [0.94-0.95] | 0.83 [0.82-0.84] | 0.85 [0.83-0.86] |
| PRC-area | 0.93 [0.93-0.93]* | 0.91 [0.91-0.91] | 0.86 [0.85-0.87] | 0.71 [0.69-0.73] |
| F1-score | 0.96 [0.96-0.96]* | 0.95 [0.95-0.95] | 0.89 [0.87-0.9] | 0.79 [0.78-0.8] |
| Specificity | 0.95 [0.95-0.96] | 0.97 [0.97-0.97]** | 0.92 [0.91-0.92] | 0.94 [0.94-0.95] |
| NPV | 0.98 [0.97-0.98]* | 0.94 [0.94-0.94] | 0.95 [0.95-0.96] | 0.83 [0.82-0.84] |
| PRC-area | 0.95 [0.94-0.96] | 0.96 [0.96-0.96]** | 0.94 [0.93-0.94] | 0.88 [0.87-0.89] |
| F1-score | 0.96 [0.96-0.97]* | 0.96 [0.96-0.96] | 0.94 [0.93-0.94] | 0.88 [0.88-0.89] |

95% confidence interval given in parentheses, abbreviations: AUC-ROC, area under the receiver operating characteristic curve; PPV = positive predictive value; NPV = negative predictive value; MCC, Matthews correlation coefficient; PRC-area, precision-recall-area (given for the prediction and exclusion of non-invasive ventilation); REPTree = reduced error pruning tree, *statistically significant value (p < 0.01) versus Random Forest, REPTree and multilayer perceptron, **statistically significant value (p < 0.01) versus Bayes Network, REPTree and multilayer perceptron

| **-2 Log-Likelihood** | **Cox & Snell R^2^** | **Nagelkerke R^2^** | **McFadden’s R^2^** | **Chi2** | **df** | **p** |
| --- | --- | --- | --- | --- | --- | --- |
| 804.75 | 0.35 | 0.49 | 0.34 | 409.72 | 11 | <0.01* |

**Supplementary Table 10:** Results of the logistic regression analysis based on the attributes of the decision tree model (threshold = 0.5, ten-fold cross-validation in WEKA)

|  | **Coefficient B** | **Standard error** | **z** | **p** | **Odds Ratio** | **95% confidence interval** |
| --- | --- | --- | --- | --- | --- | --- |
| **Constant** | 0.52 | 1.37 | 0.38 | 0.71 |  |  |
| **Rhonchus** | -0.18 | 0.33 | 0.56 | 0.58 | 0.83 | 0.44 - 1.59 |
| **Auscultation other** | -1.56 | 0.76 | 2.05 | 0.04* | 0.21 | 0.05 - 0.93 |
| **Obstruction/gasping/apnea** | -1.47 | 0.44 | 3.33 | <0.01* | 0.23 | 0.1 - 0.55 |
| **Bronchial spasm** | 4.64 | 0.47 | 9.84 | <0.01* | 103.94 | 41.22 - 262.04 |
| **Head moderate** | 1.03 | 0.28 | 3.73 | <0.01* | 2.81 | 1.63 - 4.85 |
| **Head severe** | 1.02 | 0.22 | 4.63 | <0.01* | 2.76 | 1.8 - 4.25 |
| **Head mild** | 0.37 | 0.49 | 0.76 | 0.45 | 1.45 | 0.56 - 3.77 |
| **Age** | -0.01 | 0 | 2.98 | <0.01* | 0.99 | 0.98 - 1 |
| **Heart rate** | 0 | 0 | 0.96 | 0.34 | 1 | 0.99 - 1 |
| **Respiratory rate** | 0.05 | 0.02 | 2.23 | 0.03* | 1.05 | 1.01 - 1.09 |
| **Oxygen saturation** | -0.02 | 0.01 | 1.71 | 0.09 | 0.98 | 0.96 - 1 |

| Logistic regression model | |
| --- | --- |
| Correctness (%) | 81.06 [81.01-81.11] |
| Kappa | 0.51 [0.51-0.52] |
| MCC | 0.57 [0.57-0.57] |
| AUC-ROC | 0.82 [0.82-0.82] |
| Sensitivity | 0.47 [0.46-0.47] |
| PPV | 0.94 [0.94-0.94] |
| PRC-area | 0.76 [0.76-0.76] |
| Specificity | 0.99 [0.98-0.99] |
| NPV | 0.79 [0.78-0.79] |
| PRC-area | 0.87 [0.87-0.87] |

The analysis shows an underperformance of logistic regression based on the attributes of the decision tree. The reduction of the attributes didn`t go along with improved validation results. Reasons could be non-linear separation of attributes (e.g., heart rate or age) or multicollinearity (e. g., respiratory rate and oxygen saturation). Analysis was performed for better comparability. *statistically significant (p < 0.05) in a Z-test.

**
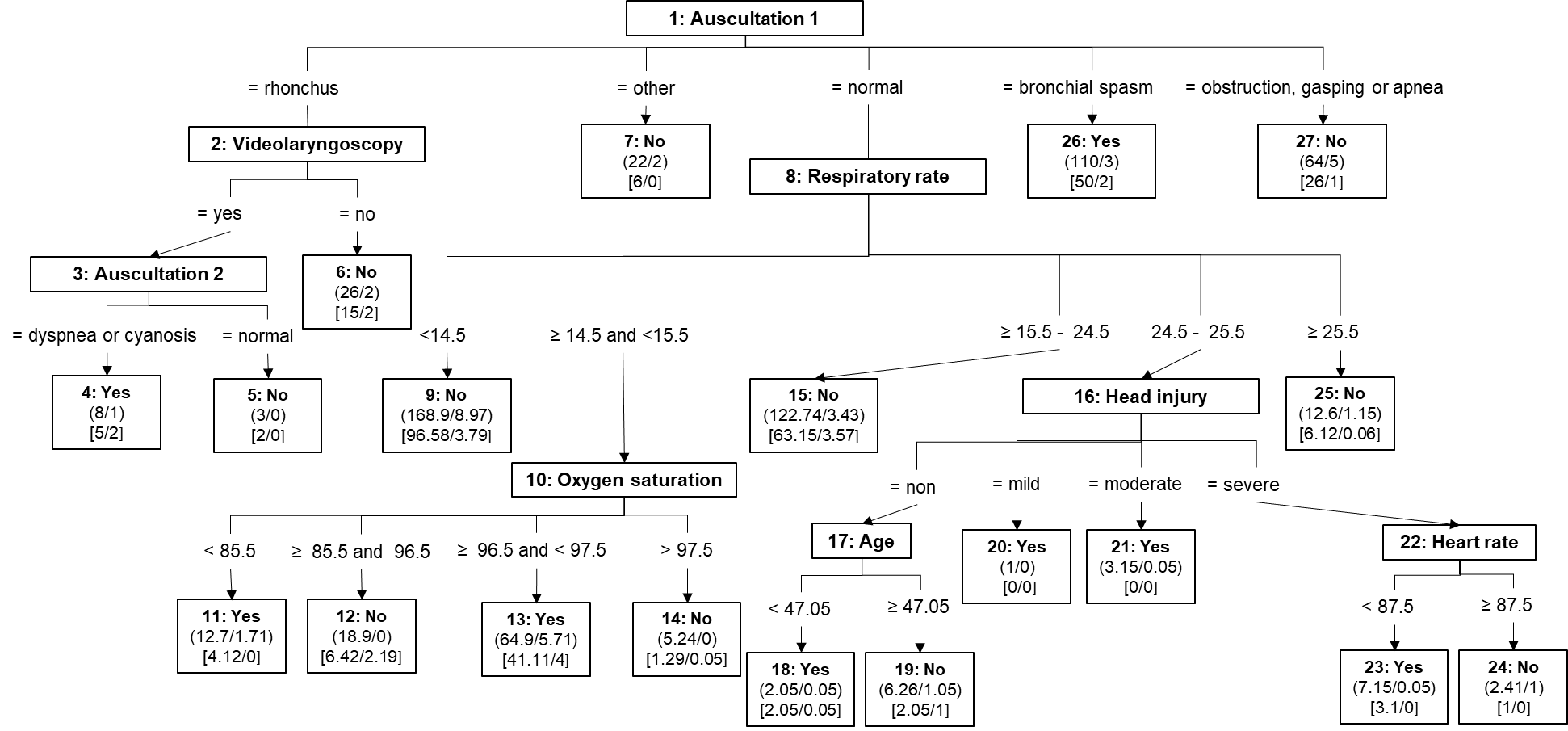
**

**Supplementary Figure 1:** REPTree (reduced error pruning decision tree) of the cross validation on the whole dataset for attribute selection with 23 nodes with 17 leaves. The tree split up at auscultation. If a bronchial spasm was diagnosed, NIV was used. In case of physiological respiratory sounds and a respiratory rate of 15/min, initial oxygen saturation was the splitting point. If it was less than 85.5% or between 96.5% and 97.5%, emergency physicians used NIV, otherwise not. If the initial respiratory rate was 25/min, non-invasive ventilation depended on the severity of head injury. NIV was used in all patients with mild or moderate head injury, if no head injury was present and they were younger than 47.05 years, or if the head was severely injured and the heart rate was below 88/min. The REPTree model had a total correctness of 91.19% (95% CI 90.75-91.63) and a sensitivity/specificity of 0.90 (95% CI 0.89-0.91) and of 0.92 (95% CI 0.91-0.92) respectively. PPV was 0.83 (95% CI 0.82-0.84) and NPV 0.95 (95% CI 0.95-0.96). It yielded an AUC-ROC of 0.93 (95% CI 0.92-0.93) and a PRC-area of 0.89 (NIV, 95% CI 0.87-0.90) and 0.94 (no NIV, 95% CI 0.93-0.94). The number in round brackets is the amount of correctly classified instances from the training set under that leaf, the second number is the number of instances which were under the leaf but had a different classification value. In the square brackets, the first number is the number of correct classifications from the pruning set and the second number is the wrong number of classifications. Non-integral number derive from averaging of the 10-times cross validation. For further information on REPTree see Witten IH, Eibe F, Hall MA, Pal CJ (2017) Data Mining: Practical Machine Learning Tools and Techniques, 4th Edition, Chapter 6 Trees and rules (https://doi.org/10.1016/C2009-0-19715-5) [18].

**Please note:** Decision trees in general are weak learners. If the training data changes, the tree changes. It is more important to draw conclusions from the used attributes and from the overall results. The division process of a decision tree can be complex and contradictory to pathophysiology or standard patient care, as tree construction is done strictly by calculation (REPTree: information gain). In a controlled study environment, this problem may not be so distinctive as data is recorded more carefully and accurately. Yet, in real world settings (like in our study), the influence on data quality is limited. Thus, estimated rather than measured values can lead to confusing results in the tree. The respiratory rate is an illustrative example of this problem: A respiratory rate of lower than 15, between 16 and 24, and 26 or greater led to different strategies in patient care compared to a rate of 15 and 25, which likely have been estimated in most patients or measured in the ECG. However, it is important to demonstrate the capabilities of machine learning algorithms not only on highly accurate study data but also on more imprecise real-world data. In order not to over-interpret the tree especially with regards to the size of the data set and the study region, we publish these results in the supplement together with this disclaimer.
